# A non-randomised controlled study to assess the effectiveness of a new proactive multidisciplinary care intervention for older people living with frailty

**DOI:** 10.1186/s12877-023-03727-2

**Published:** 2023-01-05

**Authors:** Fliss E. M. Murtagh, Mabel Okoeki, Blessing Onyinye Ukoha-kalu, Assem Khamis, Joseph Clark, Jason W. Boland, Sophie Pask, Ugochinyere Nwulu, Helene Elliott-Button, Anna Folwell, Daniel Harman, Miriam J. Johnson

**Affiliations:** 1 Wolfson Palliative Care Research Centre, Hull York Medical School, University of Hull, Hull, UK; 2 City Health Care Partnership, Hull, UK

**Keywords:** Older people, Frailty, Wellbeing, Multidisciplinary team, Integrated care, Quality of life, Proactive care

## Abstract

**Background:**

Integrated care may improve outcomes for older people living with frailty. We aimed to assess the effectiveness of a new, anticipatory, multidisciplinary care service in improving the wellbeing and quality of life (QoL) of older people living with severe frailty.

**Methods:**

A community-based non-randomised controlled study. Participants (≥65 years, electronic Frailty Index ≥0.36) received either the new integrated care service plus usual care, or usual care alone. *Data collection was at three time points: baseline, 2-4 weeks, and 10-14 weeks*. The primary outcome was patient wellbeing (symptoms and other concerns) at 2-4 weeks, measured using the Integrated Palliative care Outcome Scale (IPOS); the secondary outcome was QoL, measured using EQ-5D-5L. To test duration of effect and safety, wellbeing and QoL were also measured at 10-14 weeks. Descriptive statistics were used to characterise and compare intervention and control groups (eligible but had not accessed the new service), with t-test, Chi-Square, or Mann-Whitney U tests (as appropriate) to test differences at each time point. Generalised linear modelling, with propensity score matching, was used for further group comparisons. Data were analysed using STATA v17.

**Results:**

199 intervention and 54 control participants were recruited. At baseline, intervention and control groups were similar in age, gender, ethnicity, living status, and body mass index, but not functional status or area deprivation score. At 2-4 weeks, wellbeing had improved in the intervention group but worsened in the control (median IPOS -5 *versus* 2, *p*<0.001). QoL improved in the intervention group but was unchanged in the control (median EQ-5D-5L 0.12, *versus* 0.00, *p*<0.001). After adjusting for age, gender, and living status, the intervention group had an average total IPOS score reduction at 2-4 weeks of 6.34 (95% CI: -9.01: -4.26, *p*<0.05); this improvement was sustained, with an average total IPOS score reduction at 10-14 weeks of 6.36 (95% CI: -8.91:-3.80, *p*<0.05). After propensity score matching based on functional status/area deprivation, modelling showed similar results, with a reduction in IPOS score at 2-4 weeks in the intervention group of 7.88 (95% CI: -12.80: -2.96, *p*<0.001).

**Conclusions:**

Our findings suggest that the new, anticipatory, multidisciplinary care service may have improved the overall wellbeing and quality of life of older people living with frailty at 2-4 weeks and the improvement in wellbeing was sustained at three months.

**Ethics approval:**

NHS Research Ethics Committee 18/YH/0470 and IRAS-250981.

**Trial registration:**

The trial was retrospectively registered at the International Standard Randomised Controlled Trial Number (ISRCTN) registry (registration date: 01/08/2022, registration number: ISRCTN10613839).

## Introduction

In recent decades, healthcare for older people has been delivered by a range of providers and has sometimes been poorly coordinated between different services. Older people are increasingly living with multiple long-term conditions [1]. Integration of services is much needed and necessitates a paradigm shift in the care of older people from disease-oriented care (often focused on single conditions) towards goal-oriented proactive care (individualised and across multiple conditions) [2]. A proactive, integrated care approach that focuses on holistic health outcomes is preferable to one that focuses solely on improving individual disease outcomes [3]. The goal of an integrated care approach is to anticipate and delay the onset of poor health, as well as address existing consequences of multiple conditions, such as functional dependency and hospitalisation [4].

The UK's ageing population has resulted in an increase in the number of people living with multi-morbidity and frailty [5]. Despite this, healthcare practitioners are intent on keeping them living independently in the community, and avoiding or delaying hospitalisation [6]. Integrated care interventions should be aligned closely to the target population of older people and personalised to meet the high and diverse needs of this population [7].

In the face of these challenges, services for frail older people should be redesigned. In 2018, the Jean Bishop Integrated Care Centre in Hull was established to provide integrated, anticipatory, multidisciplinary care for older people living with frailty. This study aimed to determine whether this new, proactive, multidisciplinary care service is effective in improving the overall wellbeing and quality of life of older people living with severe frailty.

## Methods

### Study design and participants

A community-based non-randomised controlled study.

### Setting and intervention

This study was conducted within an integrated care service located in Kingston upon Hull, England, UK. The intervention group received the new integrated care service plus usual care provided by their general practitioners and other community services, while the control group received usual care only. The new integrated care service is described in Table 1.

**Table 1:** An overview of the new integrated care service according to the TIDieR checklist [8]

• The new service is an integrated, multidisciplinary, anticipatory care service provided to people identified as being at risk of moderate or severe frailty in a purpose-built community clinic (the Jean Bishop Centre).	
• Studies have shown that integrated care services improve coordination of care and health outcomes in older people living with frailty [2, 3, 7].	
• The service is provided by a specialised multidisciplinary team of geriatricians, nurse practitioners, general practitioners with an extended role in frailty care, pharmacists, occupational therapists, physiotherapists, social workers, clinical support workers, carers’ support, and volunteers.	
• A member of the team visits the patient in their home prior to the Centre attendance to pre-assess and identify concerns that the patient wishes to discuss when they attend their assessment.	
• The new service then provides various individually-tailored assessments and interventions during a single appointment, taking approximately 3-5 hours.	
• Interventions are based on the individual’s comprehensive geriatric assessment and individualised care needs. Precise contents of the intervention can be found in Supplementary Table 1.	
• All participants received personalised care planning, physical health review, assessment of psychological wellbeing/mental health, medication review, social needs review, and functional/therapy review.	
• Participants were encouraged to discuss the ReSPECT (Recommended Summary Plan for Emergency Care and Treatment)^a^ form, a tool completed by professionals to promote advance care planning and individualised recommendations for a person’s clinical treatment. Further details of the advance care planning discussions and decisions can be found in Supplementary Table 2.	
• Participants were provided with a complimentary lunch and free transport to and from the centre.	
• This study did not provide the intervention but only assessed the effectiveness of this new service on wellbeing and quality of life of older people living with frailty.	

^a^More details of ReSPECT are available at https://www.resus.org.uk/respect

### Eligibility criteria

Eligible participants were people registered with local GP practices who had attended the integrated care service (intervention group) or were from local non-participating GP practices (control group), aged 65 years and above, and identified to be at risk of severe frailty (electronic Frailty Index [eFI score ≥0.36]) [9].

### Sample size

The clinically minimally important difference in our primary outcome (IPOS total score) is 4.8, with the mean (SD) for the baseline IPOS of 27.4 (9.3) [10]. To achieve 90% power at a 5% significance level, a minimum of 80 patients per group was required.

### Participant recruitment and data collection

Potential participants were informed about the study either by a member of the integrated care centre team at pre-assessment (intervention group) or by their general practitioners (control group). Interested potential participants were then approached by the research team when attending their appointment at the Integrated Care Centre (intervention group) or at home (control group). If interested and willing to participate, they provided written or witnessed verbal informed consent. Data on demographic and clinical characteristics (including functional status) were collected at baseline; data on wellbeing and quality of life were collected at baseline, 2-4 weeks, and 10-14 weeks. All data collection was undertaken between April 2019 and March 2020.

### Instruments for data collection and outcomes

The primary outcome was wellbeing at 2-4 weeks (T1), measured using the Integrated Palliative care Outcome Scale (IPOS) [10]. IPOS is a valid and reliable self-reported measure used to assess symptoms and other concerns (overall wellbeing) among those with advanced illness [10] and at risk of frailty [11, 12]. It can be reported as a total score (17 items: scoring 0-68; higher scores indicating worse wellbeing), or subscales: physical subscale (10 items: scoring 0-40); psychological (4 items: scoring 0-16); communication/practical subscale (3 items: scoring 0-12) [10] . The clinical minimally important difference in IPOS total score is 4.8, with the mean (SD) for the baseline IPOS of 27.4 (9.3) [10]. The secondary outcome was quality of life at 2-4 weeks, measured with the 5-level EuroQOL quality of life measure (EQ-5D-5L; higher scores indicating better quality of life) [13]. The EQ-5D-5L is a self-reported quality of life assessment that comprises one question for each of the five dimensions: mobility, self-care, usual activities, pain/discomfort, and anxiety/depression, plus a visual analogue scale reporting overall health status [14]. The responses can be transformed into EQ-5D-5L index and utility scores, with 0 representing death and 1 representing perfect health [14]. Wellbeing and quality of life were also measured (again using IPOS and EQ-5D-5L) at 10-14 weeks to test safety and duration of effect. Functional status, assessed by the clinical team, was measured using the Australia-modified Karnofsky Performance Status (AKPS); a brief measure of functional status validated in cancer and non-cancer conditions [15].

### Data analysis

Demographic and clinical characteristics of both groups were described and compared using descriptive statistics and t-test, Chi-Square or Mann-Whitney U test (as appropriate) to test whether baseline differences were present. Graphical displays were used to visualise the distribution and trajectories of change in the primary and secondary outcomes over time. Propensity score matching and generalised linear modelling were used for further group comparisons, using the change scores between T1 and T0 (and T2 and T0) as the dependent variable, and using propensity score matching to manage possible baseline differences between the two groups, with the control group as the reference. Data were analysed with STATA v17 [16].

### Ethical considerations

This study obtained full ethical approvals: Integrated Research and Approval System (IRAS) -250981, and National Health Service Research Ethics Committee (NHS REC) - 18/YH/0470 before commencement.

### Trial registration

The trial was retrospectively registered at the International Standard Randomised Controlled Trial Number (ISRCTN) registry (registration date: 01/08/2022, registration number: ISRCTN10613839).

## Results

### Demographic and clinical characteristics at baseline (T0)

A total of 253 participants were recruited (199 intervention; 54 control). Participant characteristics are shown in Table 2. No statistically significant differences (p>0.05) were detected in age, gender, body mass index, ethnicity, and living status. However, compared with the control group, intervention group participants were from more deprived areas (median IMD decile 3 versus 7, *p*<0.001) but had better functional status (median AKPS 70 versus 50, *p*<0.001).

**Table 2:** Demographic and clinical characteristics of participants at baseline (T0)

	Intervention group (***N=***199)	Control group (***N=***54)	***P***-value^**a**^
**Age**			
Median (IQR)	81 (75 to 85)	82 (77 to 86)	
Mean ±SD	80 ±7	81 ±7	0.350
Min - max	65 to 99	65 to 97	
Missing (%)	0 (0.0)	3 (5.6)	
**Gender**			
Male	82 (41.2)	28 (51.8)	0.162
Female	117 (58.8)	26 (48.2)	
Missing (%)	0 (0.0)	0 (0.0)	
**Body Mass Index**			
Median (IQR)	29.3 (25.4 to 33.5)	28.4 (27.7 to 37.4)	
Mean ±SD	29.7 ±6.7	30.9 ±6.4	0.275
Min - max	15.2 to 53.8	20.4 to 46.2	
Missing (%)	5 (2.5)	4 (7.4)	
**Ethnicity**			
White	171 (89.5)	52 (96.4)	0.177 ^b^
Others	20 (10.5)	2 (3.6)	
Missing (%)	8 (4.0)	0 (0.0)	
**Living alone**			
No	106 (53.8)	27 (54.0)	0.981
Yes	91 (46.2)	23 (46.0)	
Missing (%)	2 (1.0)	4 (7.4)	
**IMD**^**c**^ **decile**			
1 (most deprived)	72 (36.2)	0	
2	19 (9.6)	0	
3	16 (8.0)	2 (3.7)	
4	27 (13.6)	1 (1.8)	
5	19 (9.6)	1 (1.8)	
6	9 (4.5)	15 (27.8)	
7	8 (4.0)	10 (18.5)	
8	6 (3.0)	12 (22.2)	
9	10 (5.0)	4 (7.4)	
10 (least deprived)	13 (6.5)	9 (16.7)	
Median (IQR)	3 (1 to 5)	7 (6 to 8)	<0.001*
Missing (%)	0 (0.0)	0 (0.0)	
**AKPS**			
40	0	16 (29.6)	
50	38 (19.1)	36 (66.7)	
60	60 (30.2)	2 (3.7)	
70	44 (22.1)	0	
80	44 (22.1)	0	
90	13 (6.5)	0	
Median (IQR)	70 (60 to 80)	50 (40 to 50)	<0.001*
Missing (%)	0 (0.0)	0 (0.0)	

^a^*p*-value of: t-test for comparing means & SDs, Mann-Whitney test for comparing medians &IQRs, and Chi-square for categorical variables

^b^Fisher’s Exact test

^c^Index of multiple deprivations [17]

### Wellbeing and quality of life at baseline (T0)

IPOS scores were similar at baseline; both in the total score (mean 19.1 *versus* 18.0, *p=*0.466), and in the physical, psychological, and communication/practical IPOS subscales (Table 3). Baseline median EQ-5D-5L index values were similar between the groups (median 0.57 *versus* 0.62, *p=*0.141) (Table 3), although significant difference in the mean EQ-5D-5L index values was detected (mean 0.53 versus 0.61, *p=*0.036) (Table 3).

**Table 3:** Wellbeing and quality of life at baseline (T0)

	Intervention group (***N=***199)	Control group (***N=***54)	*P*-value^**a**^
**Total IPOS score at T0**			
Median (IQR)	18 (10 to 26)	18 (13 to 22)	0.633
Mean ±SD	19.1 ±10.1	18.0 ±6.3	0.466
Min - max	0 to 46	9 to 38	
Missing (%)	9 (4.5)	0 (0.0)	
**Physical IPOS score at T0**			
Median (IQR)	10 (6 to 15)	10 (8 to 14)	0.507
Mean ±SD	10.7 ±6.1	11 ±4.2	0.728
Min - max	0 to 30	4 to 26	
Missing (%)	8 (4.0)	0 (0.0)	
**Psychological IPOS score at T0**			
Median (IQR)	4 (2 to 8)	4 (2 to 6)	0.503
Mean ±SD	5.1 ±3.8	4.3 ±2.6	0.198
Min - max	0 to 16	1 to 10	
Missing (%)	3 (1.5)	0 (0.0)	
**Communication/practical IPOS score at T0**			
Median (IQR)	3 (0 to 5)	2.5 (2 to 3)	0.514
Mean ±SD	3.3 ±3.1	2.7 ±2.0	0.167
Min - max	0 to 12	0 to 12	
Missing (%)	1 (0.5)	0 (0.0)	
**EQ-5D-5L index values at T0**			
Median (IQR)	0.57 (0.34 to 0.74)	0.62 (0.50 to 0.74)	0.141
Mean ±SD	0.53 ±0.29	0.61 ±0.20	0.036*
Min - max	-0.28 to 1	0.04 to 1	
Missing (%)	3 (1.5)	0 (0.0)	
**EQ-5D-5L Health today score at T0**			
Median (IQR)	60 (50 to 75)	55 (45 to 70)	0.057
Missing (%)	2 (1.0)	0 (0.0)	

^a^*p*-value of: t-test for comparing means & SDs, and Mann-Whitney test for comparing medians &IQRs

*significance level at 0.05

### Primary outcome: wellbeing at 2-4 weeks (T1)

At 2-4 weeks, the mean total IPOS score reduced (representing improved wellbeing) in the intervention group, but increased (worsened) in the control group (-5 *versus* 2, *p*<0.001) (Table 4). Similarly, for the IPOS subscales, scores improved for intervention participants but improved less or worsened for control participants: physical IPOS score (-1 *versus* -0.5, *p=*0.035), psychological IPOS score (-1 *versus* 2, *p*<0.001), and communication/practical IPOS score (-2 *versus* 1, *p*<0.001). A pattern of reduction in severe/overwhelming IPOS items in the intervention group compared with no change in control was also seen (Supplementary Table 3).

**Table 4:** Primary outcome: wellbeing at 2-4 weeks (T1)

	Intervention group (***N=***199)	Control group (***N=***54)	*P*-value^**a**^
**Difference in total IPOS score between T0 & T1**			
Median (IQR)	-5 (-11 to 0)	2 (-1 to 5)	<0.001*
Mean ±SD	-5.3 ±8.2	1.8 ±4.9	<0.001*
Min – max	-32 to 14	-8 to 17	
Missing (%)	35 (17.6)	0 (0.0)	
**Difference in Physical IPOS score between T0 & T1**			
Median (IQR)	-1 (-4 to 2)	-0.5 (-2 to 2)	0.035*
Mean ±SD	-1.5 ±4.7	0 ±3.0	0.040*
Min – max	-15 to 11	-8 to 7	
Missing (%)	32 (16.1)	0 (0.0)	
**Difference in Psychological IPOS score between T0 & T1**			
Median (IQR)	-1 (-4 to 1)	2 (0 to 3)	<0.001*
Mean ±SD	-1.5 ±3.6	1.1 ±2.6	<0.001*
Min – max	-11 to 7	-7 to 6	
Missing (%)	23 (11.6)	0 (0.0)	
**Difference in Communication/practical IPOS score between T0 & T1**			
Median (IQR)	-2 (-4 to 0)	1 (-1 to 2)	<0.001*
Mean ±SD	-2.2 ±3.2	0.7 ±2.3	<0.001*
Missing (%)	23 (11.6)	8 (14.8)	

^a^*p*-value of: t-test for comparing means & SDs, and Mann-Whitney test for comparing medians &IQRs

*significance level at 0.05

negative IPOS score values represent improvement

### Secondary outcome: quality of life at 2-4 weeks (T1)

At 2-4 weeks, the EQ-5D-5L index values show significantly higher health state utility (representing better quality of life) in the intervention group compared to the control group (change of 0.12 versus 0.00, *p*<0.001) (Table 5).

**Table 5:** Secondary outcome: quality of life at 2-4 weeks (T1)

	Intervention group (***N=***199)	Control group (***N=***54)	***P***-value ^**a**^
**Difference in EQ-5D-5L index values between T0 & T1**			
Median (IQR)	0.12 (-0.01 to 0.30)	0.00 (-0.07 to 0.09)	<0.001*
Mean ±SD	0.14 ± 0.25	0.01 ± 0.18	<0.001*
Min – max	-0.69 to 0.82	-0.52 to 0.41	
Missing (%)	23 (11.6)	0 (0.0)	
**Difference in Health today score – EQ-5D-5L between T0 & T1**			
Median (IQR)	0 (-15 to 15)	0 (-5 to 10)	0.420
Missing (%)	21 (10.6)	0 (0.0)	

^a^*p*-value of: t-test for comparing means & SDs, and Mann-Whitney test for comparing medians &IQRs

*significance level at 0.05

positive EQ-5D-5L values represents improvement.

### Changes in wellbeing and quality of life at 10-14 weeks (T2)

The total IPOS score remained significantly lower in the intervention group at 10-14 weeks, (median IPOS score reduction of 4 versus control increase 2, *p*<0.001). The EQ-5D-5L index values also remained higher (better quality of life) at 10-14 weeks, but this was not statistically significant at the 5% level (0.06 *versus* -0.01, *p*<0.068) (Supplementary Table 4). Further graphical displays showing the distribution and trajectories of change in the primary outcome are shown in Supplementary Figures 1, 2, and 3, and 4.

### Propensity score matching and modelling

After adjusting for age, gender, and living status, at 2-4 weeks the intervention group had statistically and clinically improved average total IPOS scores (-6.34; 95% CI: -9.01 to -4.26, *p*<0.05), and EQ-5D-5L index values (0.12; 95% CI: 0.04 to 0.19, *p*<0.05) (Table 6). These improvements were sustained at 10-14 weeks (total IPOS: -6.36; 95% CI to-8.91: -3.80, *p*<0.05; EQ-5D-5L: 0.07; -0.01 to 0.14) (Supplementary Table 5). After propensity score matching based on functional status and area deprivation score (given the baseline differences), the modelling showed similar results: the intervention group at 2-4 weeks the IPOS score improved (-7.88; 95% CI: -12.80 to -2.96, *p*<0.001) using nearest neighbour matching (Supplementary Table 6).

**Table 6:** Regression analysis showing the effect of the intervention on the outcomes [difference in IPOS & EQ-5D-5L scores (T1 – T0)]^a^

Outcome: difference in total IPOS scores (T1 – T0)			
	Unadjusted coefficient (95% CI)	Adjusted coefficient (95% CI)	R^**2**^
**Group**			0.175
Control	1	1	
Intervention	-7.06 (-9.40 : -4.73)*	-6.34 (-9.01 : -4.26)*	
**Outcome: difference in physical IPOS scores (T1 – T0)**			
**Group**			0.065
Control	1	1	
Intervention	-1.46 (-2.80 : -0.11)*	-1.32 (-2.69 : 0.06)	
**Outcome: difference in psychological IPOS scores (T1 – T0)**			
**Group**			0.108
Control	1	1	
Intervention	-2.63 (-3.67 : -1.59)*	-2.45 (-3.53 : -1.38)*	
**Outcome: difference in communication/practical IPOS scores (T1 – T0)**			
**Group**			0.163
Control	1	1	
Intervention	-2.93 (-3.86 : -2.00)*	-2.81 (-3.76 : -1.85)*	
**Outcome: difference in EQ 5D index values (T1 – T0)**			
**Group**			0.050
Control	1	1	
Intervention	0.13 (0.06 : 0.21)*	0.12 (0.04 : 0.19)*	
^a^Adjusted for age, gender, & living status			

*significance level at 0.05

negative IPOS scores and positive EQ-5D-5L values represent improvement.

## Discussion

We evaluated the effectiveness of a new, anticipatory, multidisciplinary care service in improving the wellbeing and quality of life for older people living with severe frailty. This study showed that the new service improved wellbeing and quality of life for this study population at 2-4 weeks; the improvement in wellbeing was sustained at 3 months. We chose a short observation time because we expect the benefit from improved symptom control and additional support will have maximal effect at 2-4 weeks. The improvement in wellbeing and quality of life associated with the new integrated care service is greater than that previously reported as clinically meaningful by patients with advanced illness [10, 18].

Choosing the right primary outcome measure is important; we found greater change in wellbeing (measured with IPOS) than in quality of life (measured with EQ-5D-5L). IPOS can detect clinically meaningful changes in symptoms and other concerns over time, and is more specific to the concerns of those with advanced illness. Quality of life, in contrast, is subject to a much wider range of influences. The domains included in IPOS are those prioritised as most important by patients with advanced illness themselves [10]. In this study population, the reported symptoms and other concerns may be linked to multiple long-term conditions, the progression of those conditions, to overall deterioration in health, or to management of health conditions [10]. IPOS can be used to capture wellbeing, to reflect the effectiveness of healthcare interventions, and to indicate care quality; it has good construct validity with three underlying factors: physical symptoms, psychological symptoms, and communication/practical issues [10].

We used a Comprehensive Geriatric Assessment (CGA)-based intervention – a multi-modal screening and treatment approach that identifies the medical, psychological and functional needs of older adults [19]. Multi-modal interventions are more likely than uni-modal interventions to improve health outcomes and to decrease frailty and depression in older people [19]. Our findings are consistent with another integrated care service (multi-disciplinary team meetings) evaluation which showed reduced rates of functional decline, emergency room visits and unnecessary hospitalisation among older people [20] . Use of CGA can improve physical and cognitive function, and reduce mortality and emergency hospitalisations [21], not only for older people in the hospital setting but also those in the community setting [22, 23]. CGA has also been shown to reduce the prevalence of frailty [24] which may be one of the mechanisms explaining our sustained benefit over time. In a realist review which assessed the use of CGA in improving health-related quality of life, findings showed that the use of CGA improved patient outcomes such as physical and cognitive function, reduced mortality and emergency hospitalisations [19], not only in older people in the hospital setting but also those in the community setting [25, 26]. However, a recent review has shown that there are significant variations in the results from earlier CGA intervention studies [27], and the evidence for effectiveness is low.

### Strengths and limitations of the study

This is one of the first studies evaluating the impact of a new, anticipatory, multidisciplinary care service for older people living with frailty on wellbeing as well as quality of life. A major strength is the use of a matched control group, with propensity matching to adjust for baseline differences. This demonstrates the course of the patients’ outcomes and supports the relationship between outcomes and intervention [28–30]. However, some of the limitations need discussion. First, there were more patients recruited in the intervention group than in the control group. This reflected study limitations during data collection (during the COVID-19 lockdown) and was not planned. Our plan was to recruit an equal number of participants for both groups but unfortunately, because of COVID, this was not possible. Unequal samples in control and intervention groups are not – of themselves – problematic, unless leading to loss of power and/or unequal variance. In this instance, the imbalance was due to accessibility problems related to COVID. We therefore describe the two groups in more detail, especially in relation to functional status and area deprivation scores.

Second, the study groups had baseline differences in functional status and area deprivation scores. This may reflect sampling; those in the intervention group had – by definition – had to be mobile enough to attend the centre, while the control group included those who were house-bound (therefore with poorer functional status). GP practices (and hence areas) were included according to the roll-out of the integrated care service across the district; and selection of GP practices for control group recruitment were constrained by the roll-out (likely contributing to differences in area deprivation scores). However, propensity score matching using functional status and area deprivation scores still showed that the new service was associated with improved patients' wellbeing, and the size of this effect was clinically meaningful [10]. For clinically important questions in observational research, propensity score analysis provides an alternate approach for evaluating causal treatment effects [30–33]. Any future study should aim to recruit participants with similar baseline characteristics to reduce sampling bias. Third, this study recruited only participants with severe frailty; the service is now extended to include those at risk of moderate frailty. Future studies should be designed to recruit participants from a wider frailty group. Fourth, this was an open trial because the service was an ongoing one, hence the intervention and outcome assessments were not blinded. This could have led to information bias. Any future study should aim to blind the study outcomes.

### Research and clinical implications

This study demonstrated that selection of relevant outcome measures as well as careful timing of measurement of primary and secondary outcomes is important in evaluations of interventions in advanced illness. There is a need for wider testing of this model of care in other populations and contexts. The clinical implications for the current findings include the need to consider wider use of this model of care among this population as well as defining the implementation strategies that can help to ensure wider adoption and sustainability of the new service.

## Conclusion

This study provides insight into the benefits of an integrated care service. Our findings suggest that the new anticipatory, multidisciplinary care service may have improved the overall wellbeing and quality of life of older people living with frailty at 2-4 weeks and the improvement in wellbeing was sustained at three months. However, change in the quality of life was not maintained at three months. The effectiveness of the new integrated care service on the outcomes of frailty, such as dependency, hospitalisation and mortality, should be considered in further studies but this initial evaluation shows real promise.

## Supplementary Information

**Additional file 1.**

## Data Availability

The dataset(s) supporting the conclusions of this article are included within the article (and its additional file(s)).
