# Supplementary material for: A non-randomised controlled study to assess the effectiveness of a new proactive multidisciplinary care intervention for older people living with frailty
Source: BMC Geriatr. 2023 Jan 5;23:6. doi: 10.1186/s12877-023-03727-2 (PMC9813451; doi:10.1186/s12877-023-03727-2)
Supplement: Supplementary file 1 — Additional file 1. [file 12877_2023_3727_MOESM1_ESM.docx]

**Supplementary material**

**Supplementary table 1**: Summary of components delivered to the intervention group (N = 199; note that most participants received several interventions)

| **Components** | **Some examples** | **N** | **%** |
| --- | --- | --- | --- |
| Physical assessment | Physical health, nutrition, oral health, bowel function, bladder function, bone health, falls risk, pain assessment, ear care, eye care, sleep | 199 | 100.0% |
| Functional/ Mobility/ Balance assessment | Mobility, lifestyle activities, wheelchair assessment, including cognitive aspects as impacting on function | 199 | 100.0% |
| Psychological/ Mental assessment | Mood, cognition, psychological health, referral for bereavement counselling, etc  (Of whom: more detailed psychological assessment) | 199  (33) | 100.0%  (16.6%) |
| Social / environmental / economic assessment | Social functioning, support, disabled facilities grant, Department of Work and Pensions advice, Hull Council information, domestic cleaning and support advice | 199 | 100.0% |
| Medication assessment and advice | Review of medication / advice on medications / changes to medication, advice on inhaler techniques | 199 | 100.0% |
| Personalised care planning | Tailored and individualized plan according to holistic health and social care needs | 199 | 100.0% |
| Consideration of future care | Offered opportunity to consider future care  (Of whom; completed ReSPECT form* - see Table 2) | 199  (95) | 100.0%  (47.7%) |
| Referral to clinics and other professionals | Various clinic referrals, dietician, physiotherapist, occupational therapist, etc - see Table 2) | 90 | 45.2% |
| Investigations | Blood tests, X-rays, Endoscopy | 53 | 26.6% |

***More details of ReSPECT are available at** [**https://www.resus.org.uk/respect**](https://www.resus.org.uk/respect)

**Supplementary table 2:** Summary of advance care planning discussions and decisions delivered to the intervention group (N = 199; participants often chose more than one decision or option)

|  | | **N** | **%** |
| --- | --- | --- | --- |
| **Decisions about preferred place of care** | | | |
| Hospital admission | Discussed about hospital admission | 16 | 16.8 |
|  | Decision already made for hospital admission | 37 | 39.0 |
|  | Conditional hospital admission (if essential) | 21 | 22.1 |
| Supportive care in usual place of residence | | 21 | 22.1 |
| **Decisions for or against specific care or treatments** | | | |
| Not for Cardiopulmonary Resuscitation | | 53 | 55.8 |
| Not for Cardiopulmonary Resuscitation or High Dependency Unit/Intensive Care Unit | | 36 | 37.9 |
| For Cardiopulmonary Resuscitation and active treatment | | 31 | 32.6 |
| For Supportive care only | | 5 | 5.3 |
| For Cardiopulmonary Resuscitation but consider deactivation of Implantable Cardioverter Defibrillator if quality of life deteriorates | | 1 | 1.1 |
| For no antibiotics | | 1 | 1.1 |
| **Decisions about who or when to decide** | | | |
| Health professionals to decide if Cardiopulmonary Resuscitation and Intensive Care unit appropriate | | 1 | 1.1 |
| Discuss or decide when acutely unwell | | 4 | 4.2 |
|  | | | |

**Supplementary table 3:** Individual IPOS and EQ-5D-5L scores for both intervention and control groups, at baseline (T0) and follow-up (T1 & T2)

|  | **Intervention group** | | | **Control group** | | | | |
| --- | --- | --- | --- | --- | --- | --- | --- | --- |
|  | **T0** | **T1** | **T2** | **T0** | **T1** | | **T2** | |
| **IPOS** |  |  |  |  |  | |  | |
| **1. Pain** | **N= 197** | **N= 178** | **N= 162** | **N=54** | | **N=54** | | **N=47** |
| Not at all | 40 (20.3) | 51 (28.6) | 34 (21.0) | 14 (26.0) | | 7 (12.9) | | 7 (14.9) |
| Slightly | 30 (15.2) | 19 (10.7) | 18 (11.1) | 12 (22.2) | | 17 (31.5) | | 7 (14.9) |
| Moderate | 63 (32.0) | 68 (38.2) | 67 (41.4) | 16 (29.6) | | 15 (27.8) | | 20 (42.5) |
| Severe | 58 (29.4) | 38 (21.4) | 43 (26.5) | 12 (22.2) | | 15 (27.8) | | 13 (27.7) |
| Overwhelming | 6 (3.1) | 2 (1.1) | 0 (0.0) | 0 (0.0) | | 0 (0.0) | | 0 (0.0) |
| **2. Shortness of breath** | **N= 197** | **N= 79** | **N= 163** | **N=54** | | **N=54** | | **N=47** |
| Not at all | 79 (40.1) | 72 (40.2) | 65 (39.9) | 15 (27.8) | | 15 (27.8) | | 13 (27.7) |
| Slightly | 40 (20.3) | 51 (28.5) | 45 (27.6) | 6 (11.1) | | 13 (24.1) | | 4 (8.5) |
| Moderate | 44 (22.3) | 47 (26.3) | 50 (30.7) | 23 (42.6) | | 19 (35.2) | | 22 (46.8) |
| Severe | 26 (13.2) | 9 (5.0) | 3 (1.8) | 10 (18.5) | | 7 (12.9) | | 8 (17.0) |
| Overwhelming | 8 (4.1) | 0 (0.0) | 0 (0.0) | 0 (0.0) | | 0 (0.0) | | 0 (0.0) |
| **3. Weakness/ lack of energy** | **N= 196** | **N= 179** | **N= 163** | **N=54** | | **N=54** | | **N=47** |
| Not at all | 51 (26.0) | 30 (16.8) | 15 (9.2) | 1 (1.8) | | 2 (3.7) | | 3 (6.4) |
| Slightly | 30 (15.3) | 34 (19.0) | 25 (15.3) | 13 (24.1) | | 8 (14.8) | | 11 (23.4) |
| Moderate | 64 (32.7) | 99 (55.3) | 111 (68.1) | 36 (66.7) | | 39 (72.2) | | 28 (59.6) |
| Severe | 39 (19.9) | 16 (8.9) | 12 (7.4) | 4 (7.4) | | 5 (9.3) | | 5 (10.6) |
| Overwhelming | 12 (6.1) | 0 (0.0) | 0 (0.0) | 0 (0.0) | | 0 (0.0) | | 0 (0.0) |
| **4. Nausea** | **N= 197** | **N= 178** | **N= 163** | **N=54** | | **N=54** | | **N=47** |
| Not at all | 166 (84.3) | 162 (91.0) | 146 (89.6) | 52 (96.4) | | 47 (87.0) | | 42 (89.4) |
| Slightly | 17 (8.6) | 12 (6.7) | 8 (4.9) | 1 (1.8) | | 3 (5.6) | | 2 (4.2) |
| Moderate | 8 (4.1) | 4 (2.3) | 9 (5.5) | 1 (1.8) | | 4 (7.4) | | 3 (6.4) |
| Severe | 6 (3.0) | 0 (0.0) | 0 (0.0) | 0 (0.0) | | 0 (0.0) | | 0 (0.0) |
| Overwhelming | 0 (0.0) | 0 (0.0) | 0 (0.0) | 0 (0.0) | | 0 (0.0) | | 0 (0.0) |
| **5. Vomiting** | **N= 198** | **N= 179** | **N= 163** | **N=54** | | **N=54** | | **N=47** |
| Not at all | 186 (94.0) | 170 (95.0) | 157 (96.3) | 54 (100.0) | | 53 (98.2) | | 42 (89.4) |
| Slightly | 5 (2.5) | 5 (2.8) | 1 (0.6) | 0 (0.0) | | 0 (0.0) | | 3 (6.4) |
| Moderate | 3 (1.5) | 4 (2.2) | 5 (3.1) | 0 (0.0) | | 1 (1.8) | | 2 (4.2) |
| Severe | 4 (2.0) | 0 (0.0) | 0 (0.0) | 0 (0.0) | | 0 (0.0) | | 0 (0.0) |
| Overwhelming | 0 (0.0) | 0 (0.0) | 0 (0.0) | 0 (0.0) | | 0 (0.0) | | 0 (0.0) |
| **6. Poor appetite** | **N= 198** | **N= 179** | **N= 163** | **N=54** | | **N=54** | | **N=47** |
| Not at all | 118 (59.6) | 100 (55.9) | 91 (55.9) | 31 (57.4) | | 28 (51.8) | | 22 (46.8) |
| Slightly | 31 (15.7) | 42 (23.4) | 22 (13.5) | 10 (18.5) | | 11 (20.4) | | 8 (17.0) |
| Moderate | 34 (17.2) | 32 (17.9) | 47 (28.8) | 9 (16.7) | | 12 (22.2) | | 12 (25.6) |
| Severe | 10 (5.0) | 5 (2.8) | 3 (1.8) | 3 (5.6) | | 2 (3.7) | | 4 (8.5) |
| Overwhelming | 5 (2.5) | 0 (0.0) | 0 (0.0) | 1 (1.8) | | 1 (1.9) | | 1 (2.1) |
| **7. Constipation** | **N= 197** | **N= 178** | **N= 163** | **N=54** | | **N=54** | | **N=47** |
| Not at all | 124 (62.9) | 121 (68.0) | 109 (66.9) | 34 (63.0) | | 36 (66.7) | | 31 (67.4) |
| Slightly | 20 (10.2) | 26 (14.6) | 16 (9.8) | 3 (5.6) | | 7 (12.9) | | 7 (15.2) |
| Moderate | 31 (15.7) | 27 (15.2) | 37 (22.7) | 14 (25.9) | | 10 (18.5) | | 5 (10.9) |
| Severe | 15 (7.6) | 4 (2.2) | 1 (0.6) | 2 (3.7) | | 1 (1.9) | | 3 (6.5) |
| Overwhelming | 7 (3.6) | 0 (0.0) | 0 (0.0) | 1 (1.8) | | 0 (0.0) | | 0 (0.0) |
| **8. Sore/ dry mouth** | **N= 197** | **N= 179** | **N= 163** | **N=54** | | **N=54** | | **N=47** |
| Not at all | 101 (51.3) | 79 (44.1) | 69 (42.3) | 18 (33.3) | | 19 (35.2) | | 13 (28.3) |
| Slightly | 36 (18.3) | 54 (30.2) | 36 (22.1) | 16 (29.6) | | 18 (33.3) | | 20 (43.5) |
| Moderate | 34 (17.3) | 42 (23.5) | 54 (33.1) | 13 (24.1) | | 15 (27.8) | | 11 (23.9) |
| Severe | 19 (9.6) | 4 (2.2) | 4 (2.5) | 7 (13.0) | | 2 (3.7) | | 2 (4.3) |
| Overwhelming | 7 (3.5) | 0 (0.0) | 0 (0.0) | 0 (0.0) | | 0 (0.0) | | 0 (0.0) |
| **9. Drowsiness** | **N= 198** | **N= 179** | **N= 163** | **N=54** | | **N=54** | | **N=47** |
| Not at all | 75 (37.9) | 53 (29.6) | 49 (30.1) | 8 (14.8) | | 13 (24.1) | | 7 (15.2) |
| Slightly | 39 (19.7) | 55 (30.7) | 62 (38.0) | 22 (40.7) | | 14 (25.9) | | 11 (23.9) |
| Moderate | 53 (26.8) | 67 (37.5) | 52 (31.9) | 19 (35.2) | | 25 (46.3) | | 25 (54.4) |
| Severe | 25 (12.6) | 4 (2.2) | 0 (0.0) | 4 (7.4) | | 2 (3.7) | | 3 (6.5) |
| Overwhelming | 6 (3.0) | 0 (0.0) | 0 (0.0) | 1 (1.9) | | 0 (0.0) | | 0 (0.0) |
| **10. Poor mobility** | **N= 198** | **N= 177** | **N= 163** | **N=54** | | **N=54** | | **N=47** |
| Not at all | 26 (13.1) | 5 (2.8) | 2 (1.2) | 2 (3.7) | | 2 (3.7) | | 0 (0.0) |
| Slightly | 32 (16.2) | 44 (24.9) | 20 (12.3) | 6 (11.1) | | 7 (12.9) | | 2 (4.3) |
| Moderate | 64 (32.3) | 87 (49.2) | 102 (62.6) | 33 (61.1) | | 25 (46.3) | | 27 (58.7) |
| Severe | 55 (27.8) | 36 (20.3) | 34 (20.8) | 12 (22.2) | | 19 (35.2) | | 16 (34.8) |
| Overwhelming | 21 (10.6) | 5 (2.8) | 5 (3.1) | 1 (1.9) | | 1 (1.9) | | 1 (2.2) |
| **11. Anxiety** | **N= 198** | **N= 179** | **N= 162** | **N=54** | | **N=54** | | **N=47** |
| Not at all | 96 (48.5) | 99 (55.3) | 72 (44.5) | 21 (38.9) | | 14 (25.9) | | 11 (23.9) |
| Slightly | 40 (20.2) | 29 (16.2) | 31 (19.1) | 21 (38.9) | | 11 (20.4) | | 15 (32.6) |
| Moderate | 30 (15.2) | 32 (17.9) | 46 (28.4) | 6 (11.1) | | 18 (33.3) | | 12 (26.1) |
| Severe | 25 (12.6) | 16 (8.9) | 12 (7.4) | 6 (11.1) | | 9 (16.7) | | 7 (15.2) |
| Overwhelming | 7 (3.5) | 3 (1.7) | 1 (0.6) | 0 (0.0) | | 2 (3.7) | | 1 (2.2) |
| **12. Family/friend anxiety** | **N= 197** | **N= 179** | **N= 163** | **N=54** | | **N=54** | | **N=47** |
| Not at all | 60 (30.4) | 49 (27.4) | 40 (24.5) | 17 (31.5) | | 9 (16.7) | | 10 (21.7) |
| Slightly | 37 (18.8) | 46 (25.7) | 38 (23.3) | 14 (25.9) | | 11 (20.3) | | 13 (28.3) |
| Moderate | 26 (13.2) | 41 (22.9) | 38 (23.3) | 16 (29.6) | | 17 (31.5) | | 14 (30.4) |
| Severe | 38 (19.3) | 29 (16.2) | 35 (21.5) | 5 (9.3) | | 16 (29.6) | | 8 (17.4) |
| Overwhelming | 46 (18.3) | 14 (7.8) | 12 (7.4) | 2 (3.7) | | 1 (1.9) | | 1 (2.2) |
| **13. Depression** | **N= 198** | **N= 179** | **N= 163** | **N=54** | | **N=54** | | **N=47** |
| Not at all | 99 (50.0) | 121 (67.6) | 94 (57.7) | 33 (61.1) | | 32 (59.2) | | 21 (45.7) |
| Slightly | 36 (18.2) | 34 (19.0) | 42 (25.8) | 10 (18.5) | | 10 (18.5) | | 10 (21.7) |
| Moderate | 38 (19.2) | 12 (6.7) | 24 (14.7) | 7 (13.0) | | 11 (20.4) | | 11 (23.9) |
| Severe | 15 (7.6) | 10 (5.6) | 2 (1.2) | 3 (5.6) | | 1 (1.9) | | 3 (6.5) |
| Overwhelming | 10 (5.0) | 2 (1.1) | 1 (0.6) | 1 (1.8) | | 0 (0.0) | | 1 (2.2) |
| **14. Peacefulness** | **N= 197** | **N= 179** | **N= 163** | **N=54** | | **N=54** | | **N=47** |
| Not at all | 54 (27.4) | 120 (67.0) | 107 (65.6) | 4 (7.4) | | 3 (5.6) | | 2 (4.4) |
| Slightly | 82 (41.6) | 36 (20.1) | 27 (16.6) | 31 (57.4) | | 30 (55.5) | | 27 (58.7) |
| Moderate | 29 (14.7) | 13 (7.3) | 20 (12.3) | 13 (24.1) | | 16 (29.6) | | 9 (19.6) |
| Severe | 18 (9.2) | 7 (3.9) | 5 (3.1) | 4 (7.4) | | 4 (7.4) | | 6 (13.0) |
| Overwhelming | 14 (7.1) | 3 (1.7) | 4 (2.4) | 2 (3.7) | | 1 (1.9) | | 2 (4.3) |
| **15. Sharing feelings** | **N= 198** | **N= 179** | **N= 163** | **N=54** | | **N=54** | | **N=47** |
| Not at all | 93 (47.0) | 117 (65.3) | 116 (71.2) | 14 (25.9) | | 7 (13.0) | | 4 (8.7) |
| Slightly | 38 (19.2) | 27 (15.1) | 31 (19.0) | 28 (51.8) | | 34 (63.0) | | 31 (67.4) |
| Moderate | 18 (9.1) | 17 (9.5) | 7 (4.3) | 7 (13.0) | | 8 (14.8) | | 8 (17.4) |
| Severe | 15 (7.6) | 5 (2.8) | 4 (2.4) | 4 (7.4) | | 3 (5.5) | | 1 (2.2) |
| Overwhelming | 34 (17.1) | 13 (7.3) | 5 (3.1) | 1 (1.9) | | 2 (3.7) | | 2 (4.3) |
| **16. Sharing information** | **N= 198** | **N= 178** | **N= 63** | **N=54** | | **N=54** | | **N=47** |
| Not at all | 97 (49.0) | 143 (80.4) | 140 (85.9) | 12 (22.2) | | 4 (7.4) | | 6 (13.0) |
| Slightly | 35 (17.7) | 23 (12.9) | 15 (9.2) | 22 (40.7) | | 18 (33.3) | | 20 (43.5) |
| Moderate | 17 (8.6) | 10 (5.6) | 6 (3.7) | 10 (18.5) | | 15 (27.8) | | 11 (23.9) |
| Severe | 21 (10.6) | 0 (0.0) | 2 (1.2) | 9 (16.7) | | 12 (22.2) | | 7 (15.2) |
| Overwhelming | 28 (14.1) | 2 (1.1) | 0 (0.0) | 1 (1.9) | | 5 (9.3) | | 2 (4.4) |
| **17. Practical problems** | **N= 198** | **N= 178** | **N= 163** | **N=54** | | **N=54** | | **N=47** |
| Not at all | 123 (62.1) | 155 (87.1) | 161 (98.8) | 45 (83.3) | | 47 (87.0) | | 43 (93.5) |
| Slightly | 30 (15.2) | 23 (12.9) | 2 (1.2) | 6 (11.1) | | 3 (5.6) | | 2 (4.3) |
| Moderate | 25 (12.6) | 0 (0.0) | 0 (0.0) | 2 (3.7) | | 3 (5.6) | | 0 (0.0) |
| Severe | 6 (3.0) | 0 (0.0) | 0 (0.0) | 0 (0.0) | | 1 (1.8) | | 1 (2.2) |
| Overwhelming | 14 (7.1) | 0 (0.0) | 0 (0.0) | 1 (1.9) | | 0 (0.0) | | 0 (0.0) |
| **EQ-5D-5L** |  |  |  |  |  | |  | |
| **1. Mobility** | **N= 197** | **N= 179** | **N= 163** | **N= 54** | | **N= 54** | | **N= 46** |
| Not at all | 29 (13.2) | 59 (33.0) | 36 (22.1) | 11 (20.4) | | 9 (16.7) | | 8 (17.4) |
| Slightly | 43 (21.8) | 44 (24.6) | 35 (21.5) | 7 (12.9) | | 8 (14.8) | | 10 (21.7) |
| Moderate | 57 (28.9) | 45 (25.1) | 57 (35.0) | 26 (48.1) | | 20 (37.0) | | 20 (43.5) |
| Severe | 58 (29.5) | 23 (12.8) | 30 (18.4) | 9 (16.7) | | 17 (31.5) | | 8 (17.4) |
| Overwhelming | 13 (6.6) | 8 (4.5) | 5 (3.0) | 1 (1.9) | | 0 (0.0) | | 0 (0.0) |
| **2. Self-care** | **N= 198** | **N= 179** | **N= 163** | **N= 54** | | **N= 54** | | **N= 46** |
| Not at all | 122 (61.6) | 122 (68.2) | 80 (49.1) | 23 (42.6) | | 26 (48.2) | | 13 (28.2) |
| Slightly | 32 (16.2) | 21 (11.7) | 35 (21.4) | 15 (27.8) | | 10 (18.5) | | 23 (50.0) |
| Moderate | 28 (14.1) | 21 (11.7) | 30 (18.4) | 8 (14.8) | | 12 (22.2) | | 5 (10.9) |
| Severe | 9 (4.6) | 3 (1.7) | 5 (3.1) | 7 (12.9) | | 6 (11.1) | | 5 (10.9) |
| Overwhelming | 7 (3.5) | 12 (6.7) | 13 (8.0) | 1 (1.9) | | 0 (0.0) | | 0 (0.0) |
| **3. Usual activities** | **N= 198** | **N= 179** | **N= 163** | **N= 54** | | **N= 54** | | **N= 46** |
| Not at all | 59 (29.8) | 61 (34.1) | 17 (10.4) | 6 (11.1) | | 7 (12.9) | | 1 (2.2) |
| Slightly | 43 (21.7) | 54 (30.2) | 32 (19.6) | 12 (22.2) | | 17 (31.5) | | 21 (45.6) |
| Moderate | 46 (23.2) | 36 (20.1) | 57 (35.0) | 29 (53.7) | | 22 (40.8) | | 17 (37.0) |
| Severe | 31 (15.7) | 13 (7.2) | 37 (22.7) | 6 (11.1) | | 7 (12.9) | | 7 (15.2) |
| Overwhelming | 19 (9.6) | 15 (8.4) | 20 (12.3) | 1 (1.9) | | 1 (1.9) | | 0 (0.0) |
| **4. Pain/discomfort** | **N= 198** | **N= 179** | **N= 163** | **N= 54** | | **N= 54** | | **N= 46** |
| Not at all | 41 (20.7) | 77 (43.0) | 60 (36.8) | 17 (31.5) | | 18 (33.3) | | 15 (32.6) |
| Slightly | 43 (21.7) | 37 (20.7) | 28 (17.2) | 12 (22.2) | | 10 (18.5) | | 4 (8.7) |
| Moderate | 66 (33.3) | 42 (23.5) | 54 (33.1) | 16 (29.6) | | 20 (37.1) | | 19 (41.3) |
| Severe | 41 (20.7) | 22 (12.3) | 21 (12.9) | 9 (16.7) | | 6 (11.1) | | 8 (17.4) |
| Overwhelming | 7 (3.6) | 1 (0.5) | 0 (0.0) | 0 (0.0) | | 0 (0.0) | | 0 (0.0) |
| **5. Anxiety/depression** | **N= 197** | **N= 179** | **N= 159** | **N= 54** | | **N= 54** | | **N= 46** |
| Not at all | 114 (57.9) | 140 (78.2) | 127 (79.9) | 41 (75.9) | | 38 (70.4) | | 33 (71.7) |
| Slightly | 37 (18.8) | 28 (15.7) | 16 (10.1) | 9 (16.7) | | 10 (18.5) | | 7 (15.2) |
| Moderate | 26 (13.2) | 9 (5.0) | 14 (8.8) | 3 (5.5) | | 4 (7.4) | | 4 (8.7) |
| Severe | 14 (7.1) | 2 (1.1) | 2 (1.2) | 1 (1.9) | | 2 (3.7) | | 2 (4.4) |
| Overwhelming | 6 (3.0) | 0 (0.0) | 0 (0.0) | 0 (0.0) | | 0 (0.0) | | 0 (0.0) |

**Supplementary table 4:** Wellbeing and quality of life at 10-14 weeks (T2)

|  | **Intervention group**  **(N=199)** | **Control group**  **(N=54)** | **P-value ^a^** |
| --- | --- | --- | --- |
| **Difference in total IPOS score between T0 & T2** |  |  |  |
| Median (IQR) | -4 (-10 to 1) | 2 (-2 to 5) | **<0.001*** |
| Mean ±SD | -4.7 ±7.9 | 2.0 ±6.6 | **<0.001*** |
| Min – max | -33 to 11 | -12 to 8 |  |
| Missing (%) | 45 (22.6) | 8 (14.8) |  |
| **Difference in Physical IPOS score between T0 & T2** |  |  |  |
| Median (IQR) | -1 (-4 to 2) | -0.5 (-2 to 4) | **0.036*** |
| Mean ±SD | -0.7 ±4.7 | 0.9 ±4.2 | 0.079 |
| Min – max | -15 to 10 | -9 to 10 |  |
| Missing (%) | 43 (21.6) | 8 (14.8) |  |
| **Difference in Psychological IPOS score between T0 & T2** |  |  |  |
| Median (IQR) | -1 (-4 to 1) | 1 (-1 to 2) | **0.003*** |
| Mean ±SD | -1.2 ±3.5 | 0.8 ±3.5 | **0.001*** |
| Min – max | -11 to 8 | -5 to 11 |  |
| Missing (%) | 38 (19.1) | 8 (14.8) |  |
| **Difference in Practical IPOS score between T0 & T2** |  |  |  |
| Median (IQR) | -2 (-5 to 0) | 0 (-1 to 1) | **<0.001*** |
| Mean ±SD | -2.8 ±3.1 | 0.2 ±1.9 | **<0.001*** |
| Min - max | -12 to 2 | -4 to 6 |  |
| Missing (%) | 37 (18.6) | 8 (14.8) |  |
| **Difference in EQ-5D-5L** **index values between T0 & T2** |  |  |  |
| Median (IQR) | 0.06 (-0.10 to 0.21) | -0.01 (-0.08 to 0.11) | 0.068 |
| Mean ±SD | 0.06 ±0.23 | -0.01 ±0.21 | 0.119 |
| Min - max | -0.63 to 0.69 | -0.68 to 0.41 |  |
| Missing (%) | 42 (21.1) | 8 (14.8) |  |
| **Difference in Health today score – EQ-5D-5L between T0 & T2** |  |  |  |
| Median (IQR) | -3 (-20 to 10) | 0 (-10 to 5) | 0.238 |
| Missing (%) | 13 (6.5) | 8 (14.8) |  |

^a^p-value of: t-test for comparing means & SDs, and Mann-Whitney test for comparing medians &IQRs

*significance level at 0.05

negative IPOS score & positive EQ-5D-5L values represent improvement.

**Supplementary table 5:** Regression analysis [outcome: difference in IPOS & EQ-5D-5L scores between T2 – T0]

| **Outcome: difference in total IPOS scores (T2 – T0)** | | | |
| --- | --- | --- | --- |
|  | **Unadjusted coefficient (95% CI)** | **Adjusted coefficient (95% CI)** | **R^2^** |
| **Group** |  |  | 0.168 |
| Control | 1 | 1 |  |
| Intervention | -6.68 (-9.21 : -4.16)* | -6.36 (-8.91 : -3.80)* |  |
| **Outcome: difference in physical IPOS scores (T2 – T0)** | | | |
| **Group** |  |  | 0.092 |
| Control | 1 | 1 |  |
| Intervention | -1.62 (-3.14 : -0.11)* | -1.35 (-2.87 : 0.17) |  |
| **Outcome: difference in psychological IPOS scores (T2 – T0)** | | | |
| **Group** |  |  | 0.064 |
| Control | 1 | 1 |  |
| Intervention | -2.03 (-3.18 : -0.87)* | -2.02 (-3.22 : -0.83)* |  |
| **Outcome: difference in communication/practical IPOS scores (T2 – T0)** | | | |
| **Group** |  |  | 0.169 |
| Control | 1 | 1 |  |
| Intervention | -2.97 (-3.92 : -2.03)* | -2.89 (-3.87 : -1.91)* |  |
| **Outcome: difference in EQ-5D-5L index values (T2 – T0)** | | | |
| **Group** |  |  | 0.029 |
| Control | 1 | 1 |  |
| Intervention | 0.07 (-0.01 : 0.14) | 0.07 (-0.01 : 0.14) |  |
| ^a^Adjusted for age, gender, & living alone | | | |
| **Supplementary Figure 1:** Trajectories of change in IPOS total scores | | | |

**Supplementary table 6:** Regression analysis [outcome: difference in IPOS scores between T1 – T0*] using propensity score matching, showing different estimation methods

| Estimation method | Differences between Intervention and Control groups | | |
| --- | --- | --- | --- |
|  | Intervention | Control | Effect estimate |
| T-test | N= 164 | N= 54 | -7.06 (-9.40 : -4.73)* |
| Regression analysis ^a^ | N= 164 | N= 54 | -6.60 (-8.94 : -4.25)* |
| Propensity score matching method (matching based on AKPS & IMD decile) ^b^ | N=51 | N= 54 | -7.88 (-12.80 : -2.96)* |

^a^Adjusted for age & gender and living status; ^b^ nearest neighbour matching with common support method

*significant at 0.05 level of significance

AKPS = Australia-modified Karnofsky Performance Scale

IMD = Index of Multiple Deprivation
